# Zooplankton Structure and Potential Food Web Interactions in the Plankton of a Subtropical Chain-of-Lakes

**DOI:** 10.1100/tsw.2002.171

**Published:** 2002-04-08

**Authors:** Karl E. Havens

**Affiliations:** Watershed Management Department, South Florida Water Management District, West Palm Beach, FL 33496, USA

**Keywords:** zooplankton, phytoplankton, nutrients, phosphorus, grazing, biomanipulation, phosphorus, subtropical lakes, Florida

## Abstract

This study evaluates the taxonomic and size structure of macro-zooplankton and its potential role in controlling phytoplankton in the Kissimmee Chain-of-Lakes, six shallow interconnected lakes in Florida, U.S. Macro-zooplankton species biomass and standard limnological attributes (temperature, pH, total phosphorus [TP], chlorophyll a [Chl a], and Secchi transparency) were quantified on a bimonthly basis from April 1997 to February 1999. Concentrations of TP ranged from below 50 to over 150 μg l. Peak concentrations of particulate P coincided with maximal Chl a, and in one instance a high concentration of soluble reactive P followed. The cladoceran zooplankton was dominated by small species, including Eubosmina tubicen, Ceriodaphnia rigaudi, and Daphnia ambigua. The exotic daphnid, D. lumholtzii, periodically was abundant. The copepods were strongly dominated by Diaptomus dorsalis, a species previously shown to be highly resistant to fish predation. These results, and findings of controlled experiments on a nearby lake with a nearly identical zooplankton species complement, suggest that fish predation may be a major factor structuring the macro-zooplankton assemblage. Zooplankton biomass, on the other hand, may be affected by resource availability. There was a significant positive relationship between average biomass of macro-zooplankton and the average concentration of TP among the six lakes. No such relationship existed between zooplankton biomass and Chl a, suggesting that the predominant food web in these systems may be based on bacteria-plankton, as has been documented in nearby Lake Okeechobee. All of the zooplankton taxa encountered in the Kissimmee Chain-of-Lakes (except *Mesocyclops edax*) are known bacteria grazers in Florida lakes. Phytoplankton biomass, measured as Chl a, was strongly associated with TP, both within and across lakes. Phytoplankton biomass was not associated with the biomass of zooplankton. These results, when considered in the context of nutrient-addition, zooplankton-exclosure studies on Lake Okeechobee, support the hypothesis that phytoplankton biomass in subtropical lakes is regulated by —bottom-up,“ rather than —top-down“ forces.

### INTRODUCTION

Zooplankton occupies a central position in the pelagic food web, transferring carbon and energy from primary producers to higher trophic levels, and potentially suppressing the abundance of phytoplankton. As such, zooplankton can directly affect the values of an aquatic resource both in terms of supporting fisheries and in maintaining good water quality. When zooplankton biomass is high, so too is the potential for fish production[1]. When zooplankton grazing is intense, it can reduce the abundance of phytoplankton to the degree that a —clear water phase" is produced[2,3]. The most dramatic impacts on phytoplankton occur when the zooplankton is dominated by large cladocerans, in particular, large species of *Daphnia*[4,5,6]. As a result, aquatic scientists and managers have pursued methods to —biomanipulate" the food webs of lakes, removing zooplanktivorous fish so that large *Daphnia* might become abundant and subsequently reduce the abundance of phytoplankton[7,8,9].

In temperate lakes, where nearly all of the research and application has occurred, the long-term success of biomanipulation has been debated[10,11]. In subtropical lakes, which are much less studied, the probability of successful control of phytoplankton by zooplankton is predicted to be very low[12]. This reflects the fact that (1) subtropical lakes generally do not have large-bodied members of the genus *Daphnia* in their zooplankton, and (2) the small *Daphnia* (*D. ambigua*) that do occur in these lakes display a pronounced decline in biomass during midsummer, the peak time of phytoplankton growth[13]. Controlled experiments have confirmed the lack of control of subtropical phytoplankton by the resident small-bodied zooplankton[14]. However, recent invasion[15,16,17] of subtropical lakes in the U.S. by D. lumholtzii, a larger species that reaches lengths >1 mm, could possibly alter this situation. This cladoceran is in the size range of the *Daphnia* species (*D. galeata* and *D. hyalina*) that are associated with clear-water phases in European lakes[2]. In general, *Daphnia* >1 mm body length can significantly reduce phytoplankton biomass per unit of phosphorus[18]. Because *D. lumholtzii* can tolerate high water temperatures[19], it can maintain high densities during summer months in subtropical lakes[17,20], presenting an opportunity for top-down control of phytoplankton.

The present study examines seasonal variations in the biomass, taxonomic composition, and size structure of crustacean zooplankton in six subtropical lakes. Empirical relationships between these attributes, water column nutrients, and the biomass of phytoplankton (measured as chlorophyll a) are considered, in order to examine the extent to which zooplankton grazing vs. nutrient availability might control primary production. The selected lakes include a diverse assemblage of zooplankton, covering a wide range of sizes, and periodically display peaks of *Daphnia*, including both *D. ambigua* and *D. lumholtzii*. An earlier study[17] focused on the potential interactions between these species and their responses to environmental conditions, in particular temperature. The present study provides a more holistic view of the zooplankton and the potential for phytoplankton control.

### METHODS

#### Study Sites

The six lakes occur in the headwaters of the Kissimmee River, which drains into Lake Okeechobee in south Florida, U.S. The lakes are located just to the south of the city of Orlando, at 27°58' to 28°20' N latitude and 81°12' to 81°13' W longitude. Zooplankton was sampled at six locations (Fig. 1) where the South Florida Water Management District routinely collects water samples in its regional surface water monitoring program. The lakes included in the study were Fells Cove, East Lake Tohopekaliga, Lake Tohopekaliga, Cypress Lake, Lake Hatchineha, and

**TABLE 1**
**Physical Attributes and Trophic State Parameters for Six Lakes in the Kissimmee Chain, Florida, U.S.**

| Lake | Area ($km^2$) | Depth (m) | pH[a] | SD (m) | TP (  g $l^{-1}$) | Chl a (  g $l^{-1}$) |
|---|---|---|---|---|---|---|
| Fells Cove | [b] | 2.3 ± 0.4 | 5.9 ± 0.6 | 0.8 ± 0.3 | 36 ± 11 | 3 ± 2 |
| East Tohopekaliga | 51 | 4.4 ± 0.4 | 7.1 ± 0.3 | 1.2 ± 0.4 | 31 ± 7 | 6 ± 3 |
| Tohopekaliga | 76 | 2.6 ± 0.3 | 7.5 ± 0.8 | 0.6 ± 0.2 | 60 ± 16 | 23 ± 11 |
| Cypress | 27 | 1.9 ± 0.5 | 7.2 ± 1.0 | 0.6 ± 0.1 | 84 ± 31 | 31 ± 18 |
| Hatchineha | 49 | 2.1 ± 0.4 | 6.5 ± 0.7 | 0.7 ± 0.5 | 76 ± 39 | 20 ± 19 |
| Kissimmee | 144 | 3.4 ± 0.3 | 7.3 ± 0.7 | 0.6 ± 0.2 | 52 ± 14 | 27 ± 15 |

Note: The trophic state data are given as means ± one standard deviation, and are based on the data collected from April 1997 to February 1999. SD = Secchi depth, TP = total phosphorus, and Chl a = chlorophyll a. The depth corresponds to depth at the sampling site, which is approximately maximal depth of the lake.

[a] Calculated from mean hydrogen ion concentrations.

[b] The area is considered in the total for East Tohopekaliga.

**Lake Kissimmee. The lakes (Table 1) range in size from 27 to 144 $km^2$, have mean depths of 2 to 4 m, and have variable trophic status, with average chlorophyll a (Chl α) ranging from <5 µg $l^{-1}$ in the northernmost lakes to >25 µg $l^{-1}$ in the south. Lakes at the northern end of the chain have considerable urban and residential development in their watersheds; lakes in the south have land use dominated by agriculture and natural wetlands. All of the lakes support high densities of fish[21,22] and develop dense mats of the floating exotic plant *Hydrilla verticillata* during summer. Phytoplankton taxonomic structure has not been quantified in these lakes, but zooplankton samples described below often contained large filaments of *Anabaena circinalis*, *A. limnetica*, and colonies of *Microcystis spp*. These taxa are known bloom-formers in south Florida lakes[20,23,24].**

### Sampling and Laboratory Methods

Crustacean zooplankton was sampled at approximately bimonthly intervals from April 1997 to February 1999 at the six locations, which corresponded to the deepest point in each lake (Fig. 1). Sampling of zooplankton was done in conjunction with measurements of water depth, water transparency with a 20-cm Secchi disk, water temperature, and collection of water samples for chemical analyses. Zooplankton was collected with duplicate vertical tows of a 30-cm diameter, 153-µm conical plankton net. The tows were from near the lake bottom to the water surface; the duplicate samples were combined into a single Whirl-Pak bag and preserved with 10% formalin-sucrose solution until counting. Water was collected from near the surface with a Van-Dorn bottle for analysis of TP and Chl α concentrations.

Zooplankton was processed by BSA Environmental Services (Ohio, U.S.). Animals were enumerated at 50-100X magnification with an inverted microscope, either by counting the entire sample when densities were low, or by counting aliquots representing a known percent of the sample when densities were high. In all cases, the counts included at least 400 individuals, providing a counting accuracy of >80%[25]. Population numeric densities were estimated from counts as animals per liter. From every sample, ten individuals of each crustacean species were measured to determine total body length, exclusive of setae and spines. Male and female copepods were enumerated and measured separately. Dry weight biomass (µg per animal) of each

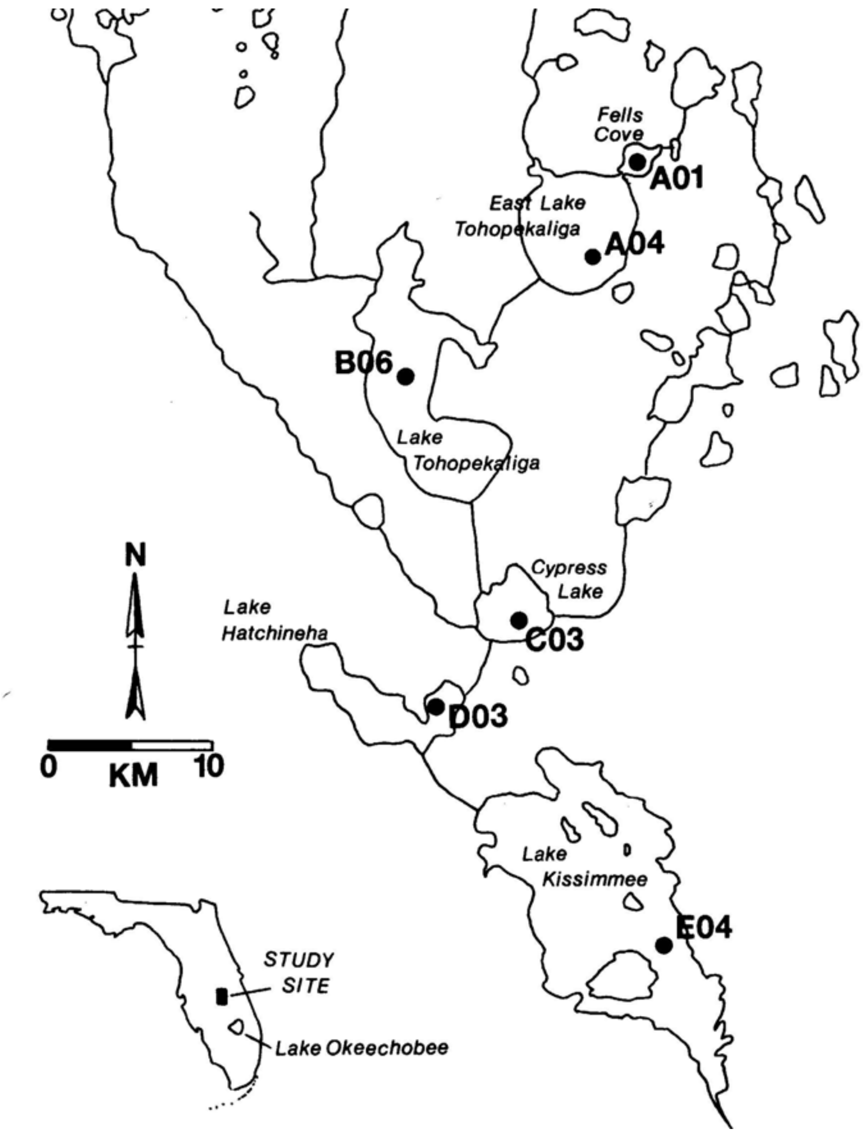

**FIGURE 1**. Map of the six study lakes and the sampling locations where data were collected in this project. The inset map shows the location in Florida, U.S. Station names (A01œE04) correspond to those used in the long-term water quality monitoring database of the South Florida Water Management District. The full water quality database can be accessed for any of these sites (period of record 1988 to present) from www.sfwmd.gov species was determined from published length-weight relationships[26]. In the case of D. lumholtzii, the equation for *D. pulex* was used, with a 15% additive term to account for the larger head and tail spines of *D. lumholtzii*. Zooplankton population biomass values (µg $l^{-1}$) were determined by multiplying numeric densities times the estimates of individual biomass.

Water chemistry analyses were performed using standard methods. Concentrations of TP were determined colorimetrically after persulfate digestion in an autoclave[27]. Soluble reactive P (SRP) was measured on undigested samples, and particulate P (PP) was determined as the difference in TP measured on filtered (0.2 µm) vs. unfiltered samples. Phytoplankton Chl a concentrations were determined according to APHA[28], after filtering samples onto Whatman GF/F filters, grinding with a tissue grinder, and extracting for 12 h in acetone in a dark freezer.

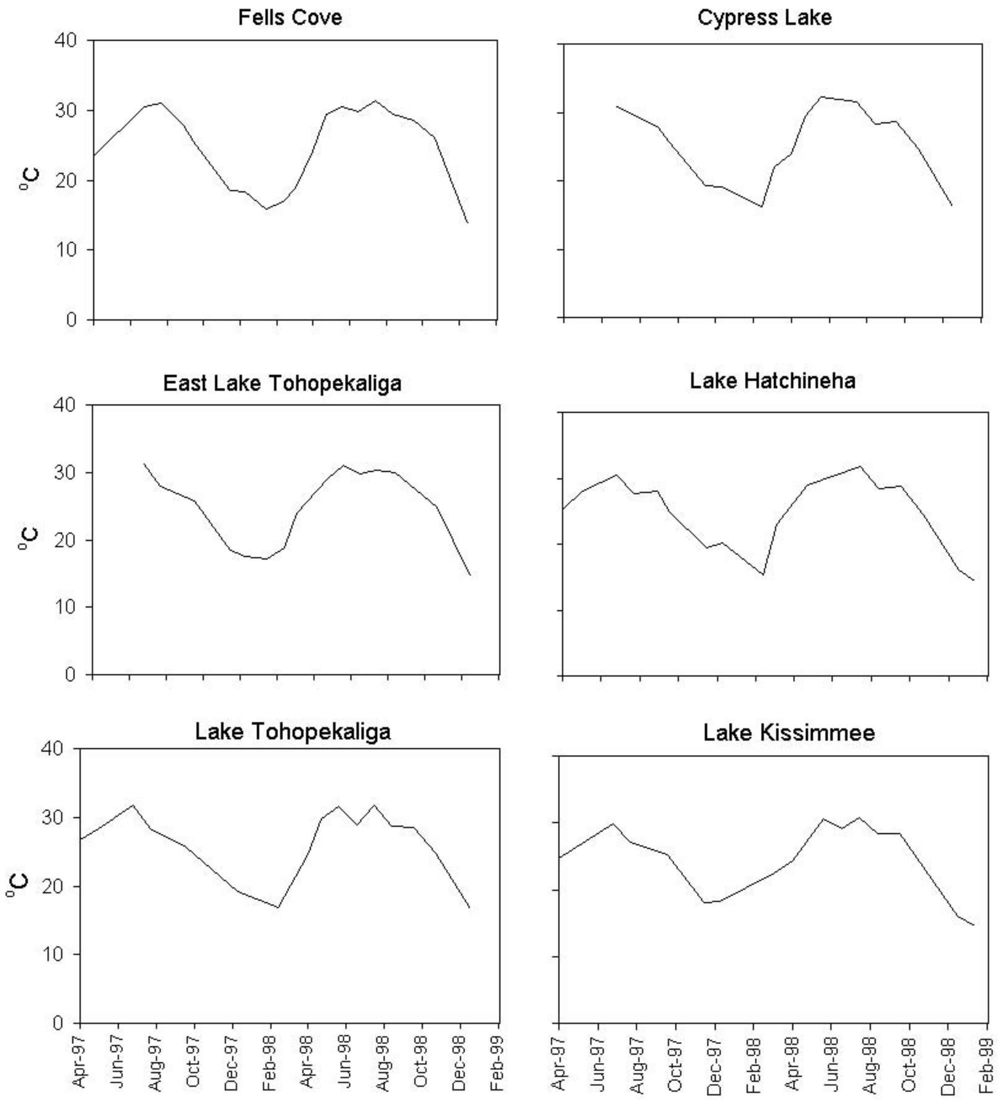

**FIGURE 2.** Near-surface water temperatures in the Kissimmee Chain-of-Lakes during 1997 and 1998.

## RESULTS AND DISCUSSION

### Physical and Chemical Conditions

Near-surface water temperatures varied from approximately 14 to 31°C, with a high degree of synchrony among lakes and similar seasonal patterns in the 2 years of sampling (Fig. 2). This degree of variation is typical of what is observed in nearby Lake Okeechobee[14] and other lakes in south Florida, and it characterizes the lakes as subtropical[29].

Concentrations of PP were highly variable among and within lakes (Fig. 3), with values consistently below 50 µg $l^{-1}$ in Fells Cove and East Lake Tohopekaliga and concentrations peaking at well over 100 µg $l^{-1}$ in Cypress Lake and Lake Hatchineha. Highest concentrations of PP occurred in June to September 1998 in all six lakes. Concentrations of SRP also varied

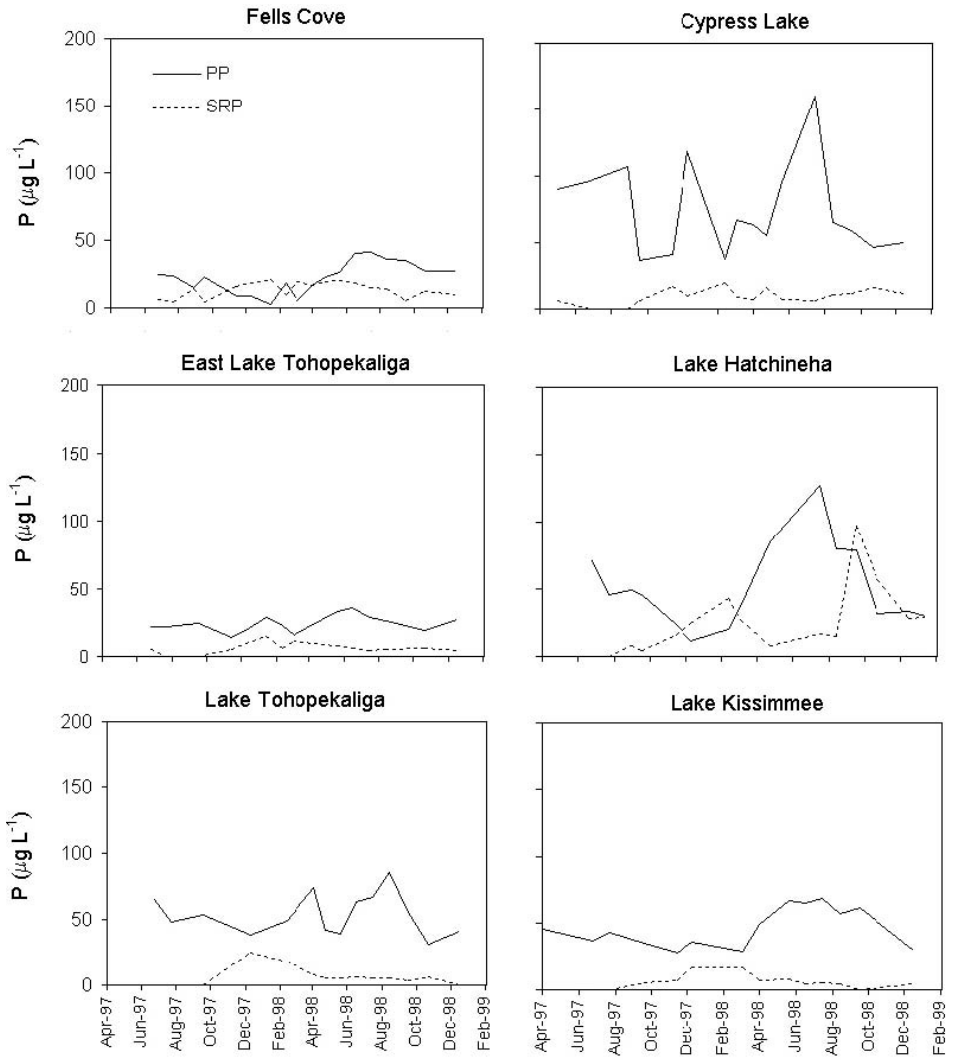

**FIGURE 3.** Near-surface concentrations of particulate phosphorus (PP) and soluble reactive phosphorus (SRP) concentrations in the Kissimmee Chain-of-Lakes during 1997 and 1998.

considerably, and there was a tendency for minimal values of SRP to coincide with maximal values of PP. There were particularly noticeable peaks in SRP just before and after the PP spike in summer 1998 in Lake Hatchineha.

### Zooplankton

Zooplankton biomass (Fig. 4) in the lakes was very low, except at times when there were synchronized short-lived peaks (see below). As indicated, the Kissimmee Chain-of-Lakes is known to support a high density of planktivorous fish[21], including gizzard shad (*Dorosoma cepedianum*) and threadfin shad (*D. petenense*). Predation by these fish might severely limit the biomass of macro-zooplankton. Bays and Crisman[30] highlighted the importance of fish,

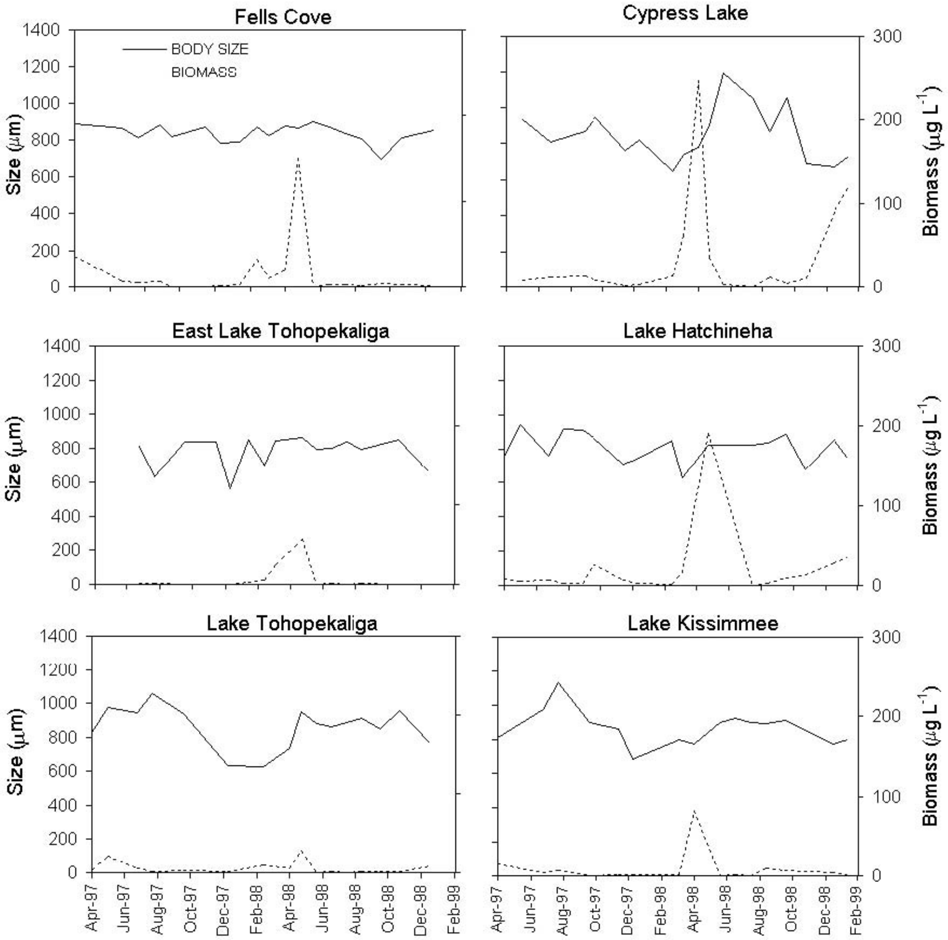

**FIGURE 4**. Average body size and total community biomass of macro-zooplankton collected in vertical net tows in the Kissimmee Chain-of-Lakes during 1997 and 1998.

especially shad, in controlling zooplankton community structure. Shad are considered to be the main determinants of macro-zooplankton biomass and body size in eutrophic Florida lakes[30,31]. When fish were experimentally removed from enclosures in nearby Lake Okeechobee, the biomass of macro-zooplankton increased more than 20-fold[12], and zooplankton survey data from that same lake strongly support the hypothesis of consumer control of that community[32].

A pronounced peak in zooplankton biomass occurred in every lake during May 1998. It is unclear what conditions led to this departure from the norm of low biomass that was observed at other times during the study. Possible explanations include relaxed fish predation and/or a short-lived increase in availability of phytoplankton or bacteria-plankton. It is noteworthy that the zooplankton biomass maxima occurred almost immediately after a period of rapidly increasing water temperature (Fig. 2) that might have stimulated growth of zooplankton food resources. A direct stimulation of zooplankton growth seems an unlikely explanation, given the lack of similar biomass maxima in the previous year when water temperatures increased in the same manner. In four of the six lakes, Chl α concentrations (see below) were increasing at the same time as zooplankton biomass. Based on research in nearby Lake Okeechobee, it is likely that bacteria-plankton biomass also was increasing, because the bacteria generally tracks changes in biomass of the phytoplankton[33]. I focus on bacteria-plankton, because much of the dominant phytoplankton in these subtropical lakes is comprised of large filamentous cyanobacteria that is not readily used as a food resource by small-bodied zooplankton[14]. All of the macro-zooplankton taxa found in the Kissimmee Chain-of-Lakes (with the exception of Mesocyclops edax) consume bacteria[34], and carbon transfer by bacteria-based pathways is very important in shallow eutrophic south Florida lakes[33,35]. In contrast to this explanation for the zooplankton maxima, the predation removal hypothesis seems less likely, given that the body size of zooplankton (see below) did not increase at the time of peak biomass. Nevertheless, experimental research is needed to elucidate the causal factors controlling zooplankton in these lakes, given its potentially important role in supporting the lake's productive recreational fishery.

The average body size of zooplankton (Fig. 4) did not display as much variation as total biomass. In Fells Cove, East Lake Tohopekaliga, and Hatchineha, average body size was relatively constant over the sampling period, at 600 to 800 μm. This small size is typical of zooplankton that is heavily impacted by fish predation[36,37,38]. In just three cases (Cypress Lake in summer 1998, Lake Tohopekaliga in summer 1997, and Lake Kissimmee in summer 1997), did the average size of zooplankton increase to over 1,000 μm.

In regard to taxonomic structure (Fig. 5, Table 2), the macro-zooplankton was dominated by calanoid copepods. *Diaptomus dorsalis* accounted for much of total biomass, while a subdominant taxon, *D. floridanus*, was less common. Small cladocerans were relatively abundant during the winter months, and there were late summer peaks of Daphnia, especially in Cypress Lake, Lake Tohopekaliga, and Lake Kissimmee. *Holopedium gibberum* and cyclopoid copepods (primarily *M. edax*) accounted for smaller portions of total biomass, except for spring and fall peaks in Lake Hatchineha. The dominant macro-zooplankton taxa in the Chain-of-Lakes (*Diaptomus dorsalis*, *D. floridanus*, *Ceriodaphnia rigaudi, Daphnia ambigua*, *D. lumholtzii*, *Eubosmina tubicen*, *H. gibberum*, and *M. edax*) are characteristic of subtropical Florida lakes[39,40,41,42,43,44]. These lakes typically display dominance by copepods and reduced densities of cladocerans in summer months. The strong dominance by *Diaptomus dorsalis* is of particular interest, and it further supports the role of fish predation in structuring the communities. Elmore[41] indicated that the distribution of *Diaptomus* species in Florida lakes is determined by resource competition and vulnerability to fish predation. *D. dorsalis* is considered to be more successful in eutrophic Florida lakes because of both its higher population growth rate and its greater ability to avoid vertebrate predators[42]. In contrast, the reduced biomass of cladocerans during summer months may be a result of stress due to high water temperature[45]. In the case of *Daphnia ambigua*, for example, fecundity is known to reach its maximum value at a water temperature of near 25°C and then decline rapidly as temperature approaches 30°C. Midsummer water temperatures in the Chain-of-Lakes were consistently in excess of 30°C. As noted by Havens et al.[17], the peak in biomass of *D. lumholtzii* in late summer in these lakes may reflect its ability to tolerate higher water temperatures than native cladocerans, as has been documented in experimental and other observational research by Work and Gophen[19,46].

### Zooplankton-Phytoplankton-Nutrient Interactions

Although this study did not include the experimental manipulations necessary to establish cause-effect relationships between zooplankton grazing, phytoplankton biomass, and nutrients, it is possible to obtain some insight from empirical relationships in the data. Based on controlled experiments performed on nearby Lake Okeechobee[12,14] with the same complement of plankton species, it can be predicted that: (1) phytoplankton biomass will be correlated with nutrients, and (2) phytoplankton will be either noncorrelated or positively correlated with the biomass zooplankton.

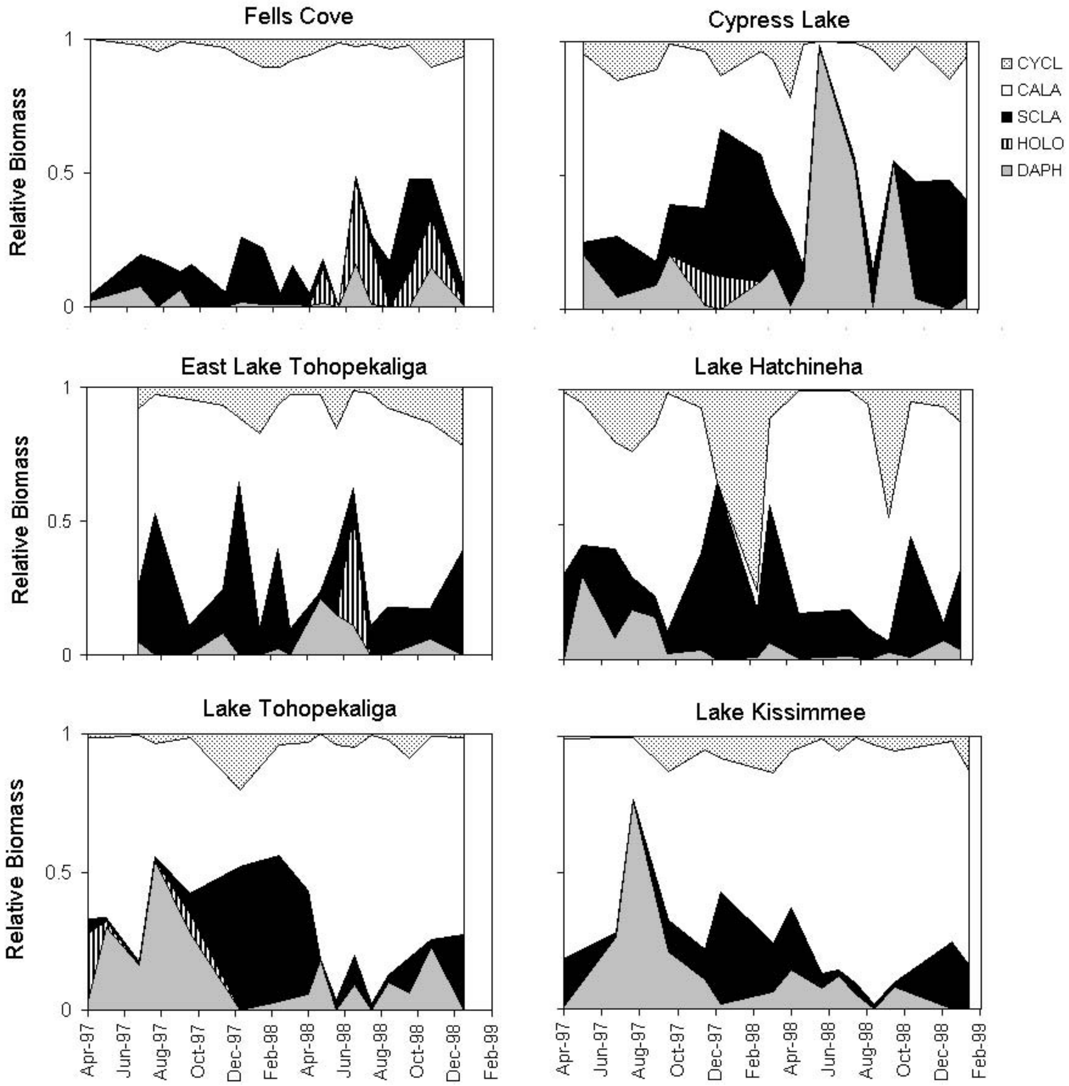

**FIGURE 5.** Relative biomass of various zooplankton taxonomic groups in the Kissimmee Chain-of-Lakes during 1997 to 1998. Taxa codes: CYCL = cyclopoid adults and copepodids; CALA = calanoid adults and copepodids; SCLA = small cladocerans; HOLO = *Holopedium gibberum*; DAPH = *Daphnia*.

The biomass of phytoplankton, measured as Chl α, was highly variable among the lakes (Fig. 6). In Fells Cove and East Lake Tohopekaliga, Chl α always was at or below 10 μg $l^{-1}$, while in the other lakes, it increased during late summer to between 40 (Lake Tohopekaliga) and 200 μg $l^{-1}$ (Cypress Lake). In general, the lakes with highest Chl α maxima also supported the highest biomass of macro-zooplankton. Fells Cove was an outlier to this pattern, with a higher average and peak biomass of zooplankton than expected for such a low concentration of Chl α. Fells Cove is a highly colored humic lake[17], and as such might support an active microbial food web based on bacteria, rather than phytoplankton[47]. In regard to the peaks in macro-zooplankton that were observed in the six lakes (Fig. 6), it is noteworthy that they occur just before or coincident with rapid increases in Chl α. Only in Lakes Tohopekaliga and Kissimmee is there evidence of a small depression in Chl α at the time of maximal biomass of macro-zooplankton. In general, these results do not indicate any strong consumer control of phytoplankton by the macro-zooplankton grazers. Plots of zooplankton biomass vs. Chl α in the six lakes (Fig. 7) indicate more clearly that

**TABLE 2**
**Relative Biomass (%) of Dominant Macro-Zooplankton Taxa in Six Lakes of the Kissimmee Chain, Florida, U.S.**

| Taxon | Fells | E Toho | Toho | Cypr | Hatch | KISSI |
|---|---|---|---|---|---|---|
| Cladocerans | | | | | | |
| Bosminopsis deitersi | 1.2 | 1.4 | 0 | 0 | 0 | 0 |
| Eubosmina tubicen | 2.4 | 5.8 | 9.5 | 17.5 | 13.8 | 9.0 |
| Ceriodaphnia rigaudi | 0 | 0.5 | 1.8 | 11.1 | 4.1 | 8.1 |
| Daphnia lumholtzii | 0.6 | 2.6 | 15.9 | 2.3 | 1.1 | 4.9 |
| D. ambigua | 1.2 | 9.8 | 0.7 | 3.1 | 1.4 | 8.9 |
| Diaphanosoma brachyurum | 1.5 | 0.5 | 0.5 | 1.1 | 0.4 | 0.2 |
| Holopedium gibberum | 7.5 | 0.3 | 1.3 | 0 | 0 | 0 |
| Copepods | | | | | | |
| Diaptomus dorsalis | 44.2 | 63.2 | 64.9 | 47.6 | 68.4 | 49.5 |
| D. floridanus | 28.7 | 0 | 0 | 0 | 0 | 4.9 |
| Mesocyclops edax | 3.2 | 2.1 | 0.7 | 11.5 | 3.6 | 3.6 |
| Calanoid copepodids | 8.2 | 10.5 | 2.9 | 3.3 | 3.1 | 9.4 |
| Cyclopoid copepodids | 1.1 | 1.6 | 0.6 | 1.0 | 1.1 | 0.7 |

Note: The taxa listed here comprised at least 1% of total macro-zooplankton biomass in at least one of the six lakes. Fells = Fells Cove, E Toho = East Lake Tohopekaliga, Toho = Lake Tohopekaliga, Cypr = Cypress Lake, Hatch = Lake Hatchineha, and Kissi = Lake Kissimmee.
Data are based on the period of record from April 1997 to February 1999.

the two attributes are noncorrelated. In contrast to the findings for zooplankton, Chl a was significantly correlated with TP in five of the lakes, including lakes with a very small (Fells Cove) and wide (Cypress Lake) range of the water quality attributes (Fig. 8). These results support the hypothesis that phytoplankton biomass is primarily controlled by resource availability, rather than zooplankton grazing, as was shown experimentally in nearby Lake Okeechobee[14].

Finally, if one plots the lake-averaged data (Fig. 9), there is a significant positive relationship between Chl a and TP, no relationship between zooplankton biomass and Chl a, and a significant positive relationship between zooplankton biomass and TP. These results support the hypothesis that a bacteria based food web is primarily supporting the macro-zooplankton production in the subtropical Florida lakes, consistent with the findings of Havens et al.[35].

### CONCLUSIONS

The results of this study provide support for hypotheses (Table 3) that previously have been stated regarding consumer vs. resource control of plankton of subtropical Florida lakes. The conclusions can be summarized in a simple conceptual model (Fig. 10), with arrows between components corresponding to strength of interaction. First, the data suggest that planktivorous fish control macro-zooplankton community structure. As indicated, the fish assemblages of these lakes contain high densities of zooplanktivores[21], including threadfin and gizzard shad. These fish have been documented to control zooplankton community structure in other lakes in Florida[30]. The observed low biomass of macro-zooplankton and the dominance by small-bodied taxa (e.g., *E. tubicen*, *Ceriodaphnia rigaudi*, and *D. ambigua*) and taxa that are well-defended against predation (*Diaptomus dorsalis*) represent the expected situation in a community controlled by fish[30,31,32].

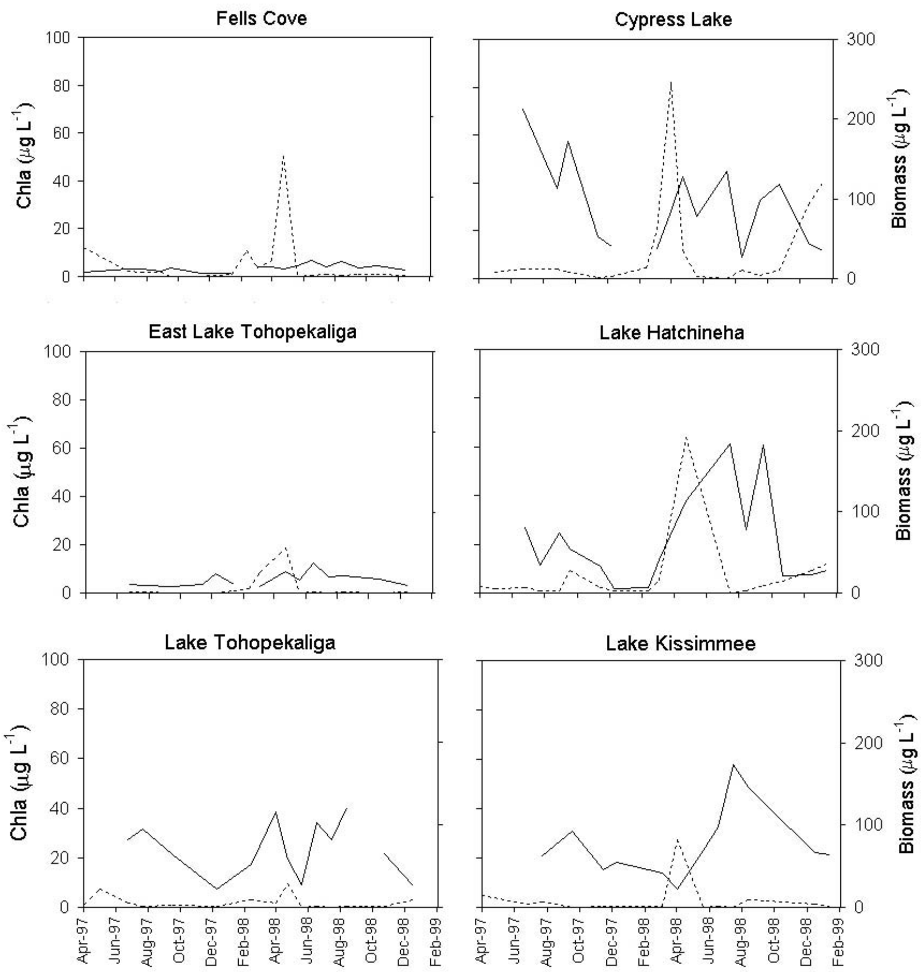

**FIGURE 6.** Near-surface concentrations of chlorophyll a (SOLID LINE, Chl a) and total community biomass of macro-zooplankton (DASHED LINE) in the Kissimmee Chain-of-Lakes during 1997 and 1998.

The lack of significant negative relationships between Chl α and zooplankton biomass is consistent with a hypothesis that zooplankton grazing has little influence on phytoplankton biomass. It is consistent with results from zooplankton exclosure studies on nearby Lake Okeechobee, where a substantial reduction in macro-zooplankton did not result in significant increases in Chl α inside experimental enclosures[14]. In contrast, there is evidence that phytoplankton are controlled by nutrient availability, based on the significant positive relationships between Chl α and TP in five of the six lakes and across lakes for the lake-averaged data. Similarly, Chl a increases were documented where nutrients were added to enclosures in Lake Okeechobee, as long as the light climate was sufficient for net algal growth[14,20]. The Kissimmee Chain-of-Lakes are more likely to be nutrient limited than Lake Okeechobee, because they are not affected by the high levels of abiotic turbidity that often occur in the larger lake system. With the exception of high levels of organic color in Fells Cove and East Lake Tohopekaliga, most of the underwater light attenuation in the Kissimmee lakes appears to be due to plankton (Havens, unpublished analysis of Chl α vs. Secchi relationships). In contrast, light attenuation in Lake Okeechobee is strongly controlled by abiotic seston[48,49].

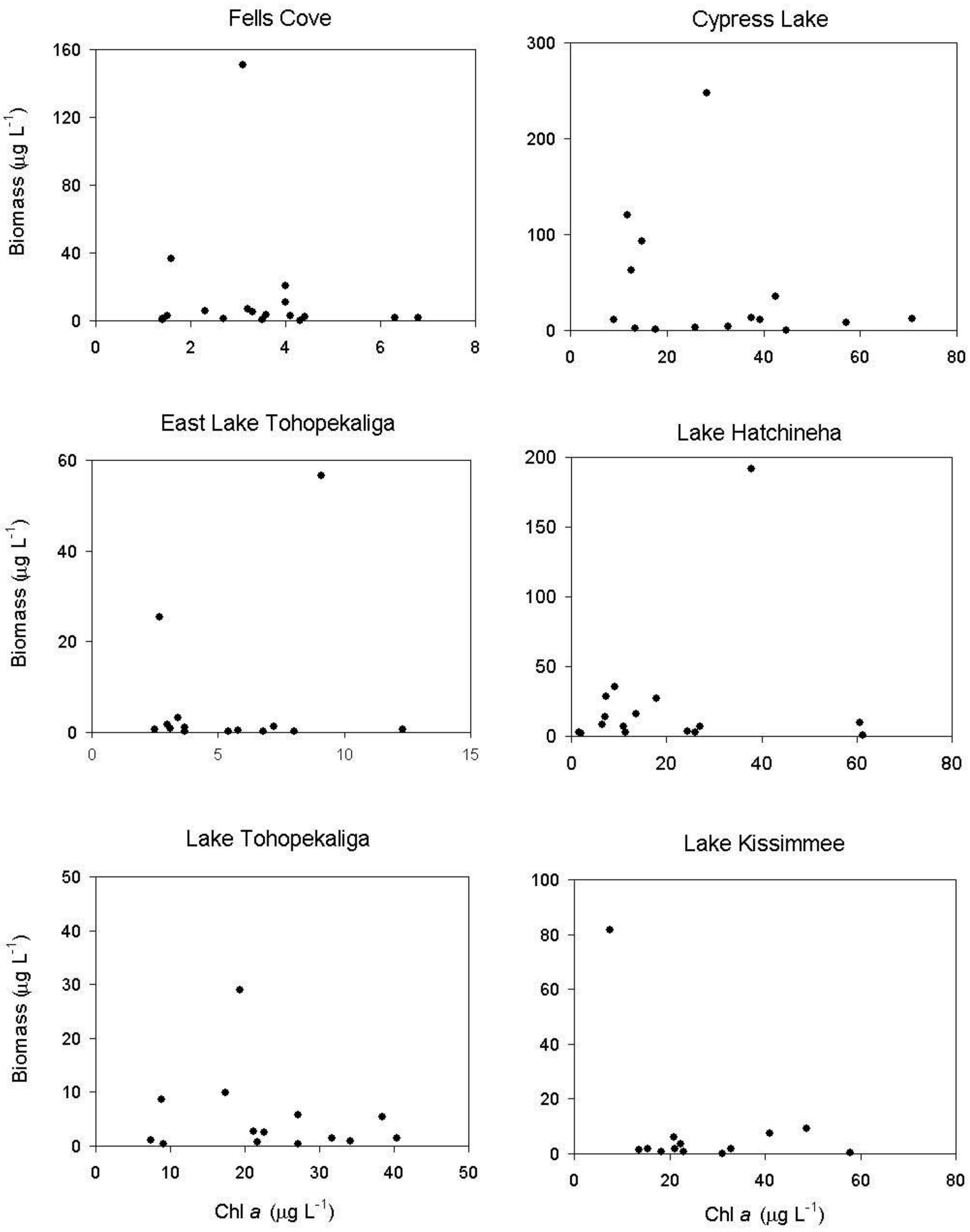

**FIGURE 7.** Community biomass of macro-zooplankton, plotted as a function of near-shore concentrations of chlorophyll a (Chl α) on each sampling event, for the six study lakes.

Although more speculative, the present results also suggest an important role for bacteria-based food webs in supporting the biomass of macro-zooplankton. In Lake Okeechobee, we have documented that this is the case[33,35], based on detailed evaluation of trophic links using radio-tracer methods. The reason that bacteria-based pathways are of such importance is that macro-zooplankton generally is small, and dominant phytoplankton is large. This situation creates a —bottle-neck“ in the traditional grazing food chain[50] (algae → zooplankton) and

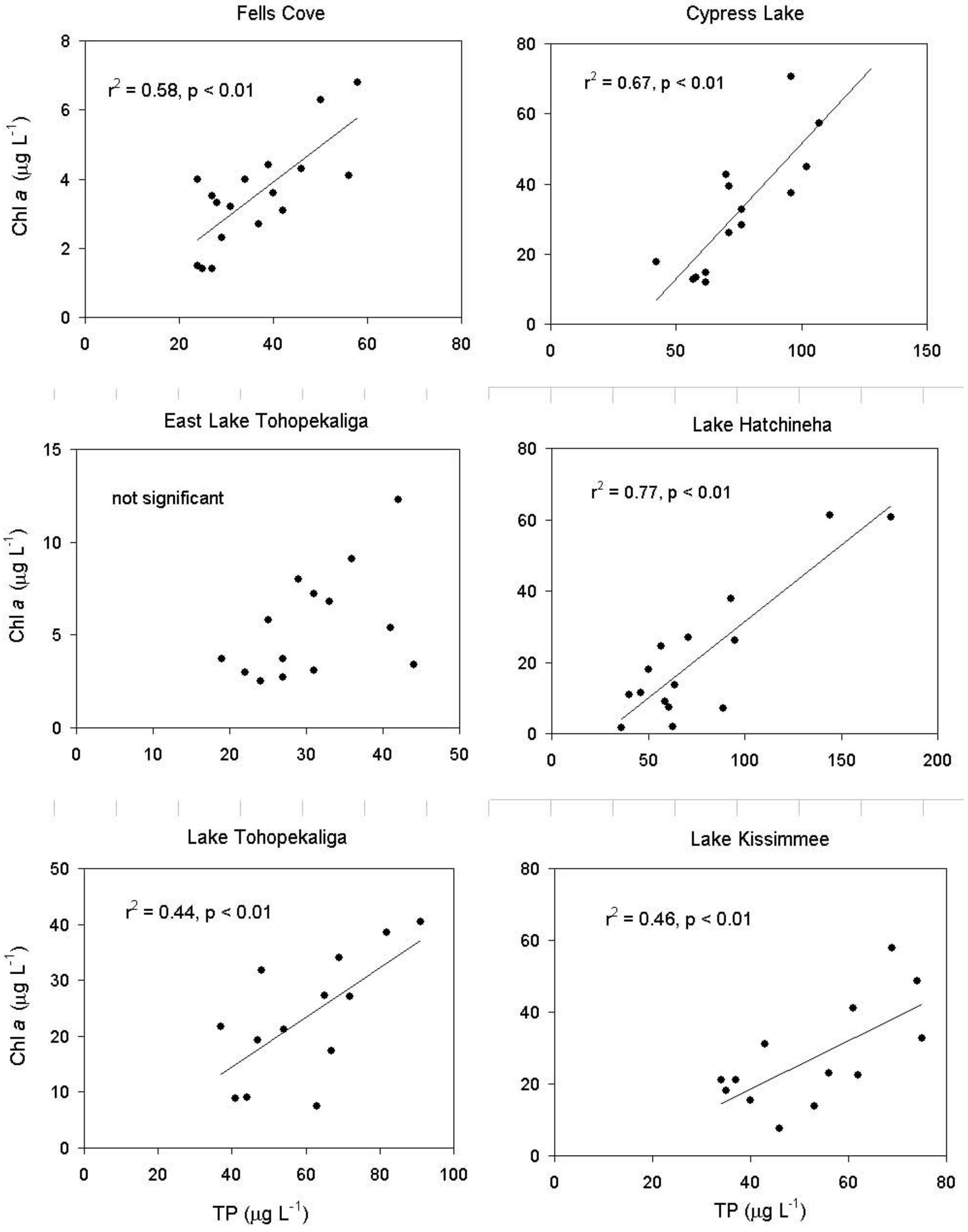

**FIGURE 8**. Near-surface concentrations of chlorophyll a (Chl α), plotted as a function of total phosphorus (TP) on each sampling event, for the six study lakes. Least-squares regression models are included where p values are <0.10.

results in zooplankton that largely are dependent on bacteria and protozoa as food resources. The finding that lake-averaged zooplankton biomass in the Kissimmee lakes is strongly related to TP, but not Chl α, supports the notion of resource control of zooplankton by something other than phytoplankton biomass. It is noteworthy that all of the macro-zooplankton taxa encountered in the Kissimmee lakes (with the exception of *M. edax*) have been documented to consume bacteria-plankton[34].

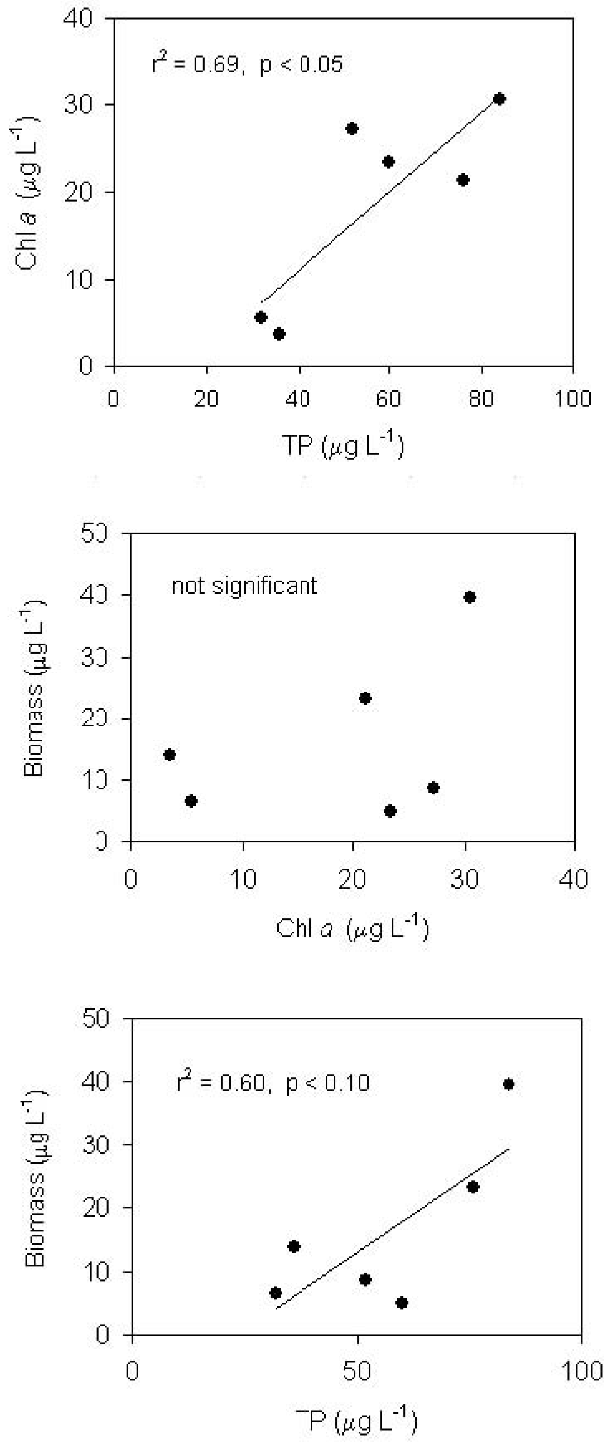

**FIGURE 9.** Relationships between lake-average (mean of all observations) epilimnetic concentrations of chlorophyll a (Chl α), total phosphorus (TP), and macro-zooplankton community biomass. Least-squares regression models are included where p values are <0.10.

Controlled experiments are a next logical step in providing causal linkages between nutrients, phytoplankton, zooplankton, and forage fish in these subtropical lakes. The results could be of value both in regard to water quality and fisheries management, given the central role of zooplankton in the pelagic ecosystem.

**TABLE 3**
**Hypotheses Regarding Consumer vs. Resource Control of Plankton in Florida Lakes, withSupport Provided by Results of the Present Study (in italics) and Other Recent Research**

| Hypothesis | Support |
|---|---|
| Intense fish predation controls macro-zooplankton community structure. | Documented high densities of zooplankton-grazing fish[21] |
| | *Dominance of small-bodied zooplankton taxa and taxa known to have strong ability to escape from vertebrate predators (Diaptomus dorsalis)* |
| | Significant changes in zooplankton composition after fish removal in a mesocosm experiment on a nearby lake with very similar plankton community[12] |
| Zooplankton grazing has minimal impacts on the biomass of phytoplanktonbiomass | *Lack of negative relationship between zooplankton biomas or size with the biomass of phytoplankton (chlorophyll a)*<br>Lack of response of phytoplankton to exclosure of zooplankton during mesocosm experiments in a nearby lake with very similar plankton community[14] |
| Nutrients control biomass of phytoplankton | *Positive relationships between total phosphorus and chlorophyll a, both within and among lakes* |
| | Positive response of phytoplankton to nutrient additions in nearby lakes when irradiance was sufficient for net growth[14,20] |
| Bacteria-based food webs control biomass of zooplankton | Positive relationship between lake-average biomass of macro-zooplankton and total phosphorus, but lack of such a relationship of zooplankton with chlorophyll a<br>Results of detailed investigations of carbon fluxes in phytoplankton vs. bacteria-based pathways in a nearby lake with very similar plankton community[33,35] |
| | Dominant zooplankton taxa in the study lakes are known to graze bacteria[34] |

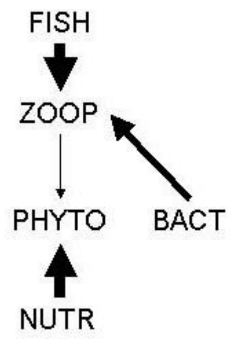

### ACKNOWLEDGEMENTS

The author is grateful to Joseph Marcus, Patrick Essex, Benjamin Bolan, and Stephanie Raymond for collecting the plankton and water quality samples, and to John Beaver, for macro-zooplankton sample processing. Peter Doering, Therese East, Susan Gray, John Beaver, and Xie Ping provided constructive comments on an earlier version of this manuscript.
